# Metal-centred azaphosphatriptycene gear with a photo- and thermally driven mechanical switching function based on coordination isomerism

**DOI:** 10.1038/ncomms14296

**Published:** 2017-02-08

**Authors:** Hitoshi Ube, Yoshihiro Yasuda, Hiroyasu Sato, Mitsuhiko Shionoya

**Affiliations:** 1 Department of Chemistry, Graduate School of Science, The University of Tokyo, 7-3-1 Hongo, Bunkyo-ku, Tokyo 113-0033, Japan; 2 Rigaku Corporation, 3-9-12 Matubara-cho, Akishima, Tokyo 196-8666, Japan

## Abstract

Metal ions can serve as a centre of molecular motions due to their coordination geometry, reversible bonding nature and external stimuli responsiveness. Such essential features of metal ions have been utilized for metal-mediated molecular machines with the ability to motion switch via metallation/demetallation or coordination number variation at the metal centre; however, motion switching based on the change in coordination geometry remain largely unexplored. Herein, we report a Pt^II^-centred molecular gear that demonstrates control of rotor engagement and disengagement based on photo- and thermally driven *cis*–*trans* isomerization at the Pt^II^ centre. This molecular rotary motion transmitter has been constructed from two coordinating azaphosphatriptycene rotators and one Pt^II^ ion as a stator. Isomerization between an engaged *cis*-form and a disengaged *trans*-form is reversibly driven by ultraviolet irradiation and heating. Such a photo- and thermally triggered motional interconversion between engaged/disengaged states on a metal ion would provide a selector switch for more complex interlocking systems.

## Abstract (web summary)

In metal-based molecular motors, the motion is generally triggered by changes in the ligand coordination around the metal centre. Here, the authors synthesize a molecular gear that switches between states through photo- and thermally driven geometrical isomerization around a platinum ion.

Transmission of rotary motion is a key process of molecular machines^1^,^2^,^3^. To correlate two or more movable elements in a controllable manner, a stator, which can bring them appropriately close to each other, is a key part of motion. A significant number of excellent examples have been reported on synthetic molecular gearing systems with intramolecularly correlated rotators^4^,^5^,^6^,^7^,^8^,^9^. However, the control of rotary transmission between molecular rotators is still in an early phase^10^,^11^, and therefore, in particular, *switchable* motion transmission is a challenge. Dynamic engagement between rotators is a typical rotary transmission in molecular machines. Triptycene is a well-known part of gear molecules with a rigid, highly symmetrical paddlewheel structure. More than one triptycene rotators can be covalently connected with an organic stator designed so as to have a proper positional relationship between the connected rotators^12^,^13^,^14^,^15^,^16^,^17^,^18^,^19^,^20^,^21^. Another unique example of covalently linked systems is a silicon-centred bistriptycene system^19^, which undergoes switchable gearing triggered by a chemical stimulus, fluorination/defluorination. We focused on metal ions as a control element of molecular motions due to their essential features such as reversible bonding natures with ligands, dynamic ligand exchange and external stimuli responsiveness^22^,^23^,^24^. A certain number of excellent examples of metal-mediated molecular machines capable of motion switching via metallation/demetallation or coordination number variation on the metal centre have been reported^25^,^26^,^27^.

Herein, we report a Pt^II^-centred molecular gear that demonstrates control of rotor engagement and disengagement based on photo- and thermally driven *cis*–*trans* isomerization at the Pt^II^ centre. This molecular rotary motion transmitter has been constructed from two coordinating azaphosphatriptycene rotators and one Pt^II^ ion as a stator. Isomerization between an engaged *cis*-form and a disengaged *trans*-form is reversibly driven by ultraviolet irradiation and heating. Such a photo- and thermally triggered motional interconversion between engaged/disengaged states on a metal ion would provide a selector switch for more complex interlocking systems.

## Results

### Design of metal-centred molecular gear

**Figure 1:**
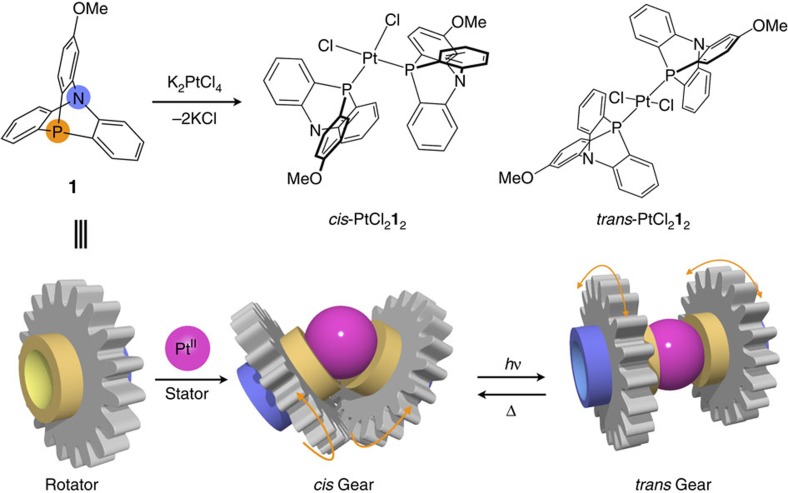
Schematic representation of a Pt^II^-centred molecular gear PtCl_2_**1**_2_. This molecular gear has two azaphosphatriptycene rotators coordinating to the central Pt^II^ ion as a stator. Isomerization between an engaged *cis*-form and a disengaged *trans*-form are reversibly driven by ultraviolet irradiation at 360 nm and heating.

In this study, we have developed a metal-centred molecular gear PtCl_2_**1**_2_, in which two ligands as rotators, 2-methoxy-9-aza-10-phosphatriptycene (**1**), directly bind to the Pt^II^ stator^28^,^29^. One striking feature of this system is a clutch-like function that allows switching of the engagement of the two rotators based on photo- and thermally driven *cis*–*trans* isomerization on the Pt^II^ centre in a traceless manner with no chemical by-products (Fig. 1)^30^,^31^.

### Synthesis of azaphosphatriptycene ligand

2-Methoxy-9-aza-10-phosphatriptycene (**1**), in which the bridgehead positions of triptycene are replaced by a nitrogen and a phosphorus atoms, was chosen as a ligand-type rotator^32^. The rotator **1** was synthesized from 3-anisidine in four steps in 9% overall yield, and was characterized by ^1^H, ^13^C and ^31^P nuclear magnetic resonance (NMR) spectroscopy, electrospray ionization-mass spectrometry and elemental analysis (Supplementary Figs 1–10). We expected that when two rotators **1** as monodentate phosphine ligands bind to a compatible Pt^II^ ion in a *cis* position, they should gear with each other.

### Preparation and characterization of Pt^II^ complexes

**Figure 2:**
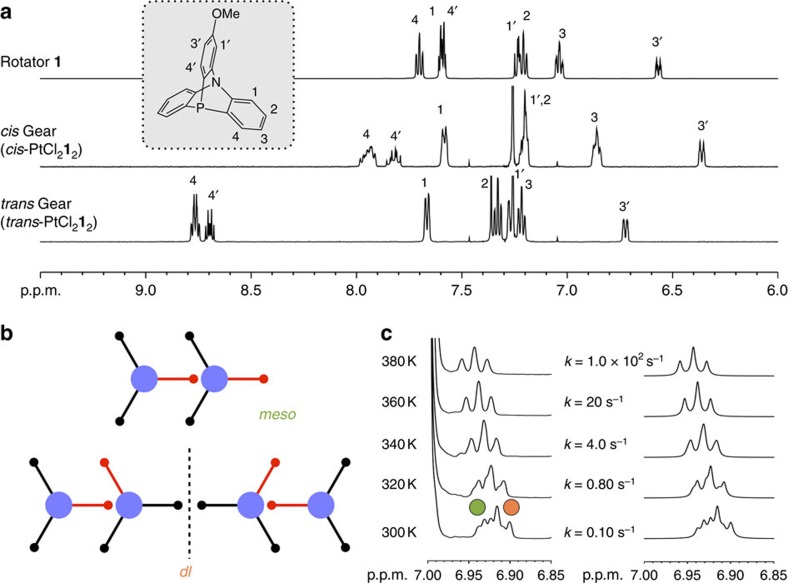
^1^H NMR spectra of rotator 1 and *cis*- and *trans*-PtCl_2_**1**_2_. The signals of a methoxy group of rotator **1** (∼3.8 p.p.m.) are omitted for clarity. For the whole NMR spectra, see the Supplementary Figures 7, 11 and 16. (**a**) ^1^H NMR spectrum of (i) **1**, (ii) *cis*-PtCl_2_**1**_2_ and (iii) *trans*-PtCl_2_**1**_2_ (500 MHz, CDCl_3_, 300 K). The ^1^H NMR spectra of *cis*-PtCl_2_**1**_2_ include *meso* and *dl* isomers in a ∼1:2 ratio. (**b**) Isomerism based on rotational conformation. (**c**) Observed and simulated spectra of 3-positions' proton at varied temperatures (500 MHz, TCE-*d*_2_/toluene-*d*_8_=1:1). Left: observed spectra in the range from 380 to 300 K. Right: simulated spectra based on the two-state exchange model. Green and orange circles denote *meso* and *dl* isomers, respectively.

The reaction of rotator **1** and 0.5 eq of K_2_PtCl_4_ in EtOH/H_2_O (1/1, v v^−1^) at room temperature for 21 h afforded a mixture of square-planar Pt^II^–phosphine complexes, *cis*-PtCl_2_**1**_2_ and a small amount of *trans*-PtCl_2_**1**_2_ (Figs 1, 2a). After recrystallization from CHCl_3_/diethyl ether, pure *cis*-PtCl_2_**1**_2_ was successfully obtained as colourless crystals in 59% yield (Supplementary Figs 11 and 13). The coordinating donor atom and the geometry around the Pt^II^ centre in solution were determined by NMR spectroscopy. In a ^31^P NMR spectrum of the *cis* isomer in C_6_D_6_, a ^31^P-^195^Pt coupling was observed (*J*_1_=3,625 Hz; Supplementary Fig. 12) due to the binding of phosphine ligand to the central Pt^II^ ion. Then, a ^1^H NMR spectrum of the *cis* isomer showed that the proton signals for the 3-positions of azaphosphatriptycene shifted upfield from the free ligand, indicating that two azaphosphatriptycene ligands were close to each other. Moreover, a lineshape analysis of the spectral pattern of the proton signals of the 4-positions suggests that there are two rotational isomers, *meso* and *dl* forms, in a 1:2 ratio in solution (Fig. 2b, Supplementary Fig. 14). We analysed the kinetics of the gear slippage during interconversion between *meso* and *dl* isomers of *cis*-PtCl_2_**1**_2_. Variable temperature ^1^H NMR measurement of *cis*-PtCl_2_**1**_2_ was described by the two-signal overlap model of the *meso* and *dl* isomers. An Eyring plot of the exchange rates of the isomers at every temperature gave activation parameters of the interconversion, Δ*H*^‡^=16.4±0.2 kcal·mol^−1^ and Δ*S*^‡^=−0.9±0.7 cal·K^−1^ (Fig. 2c, Supplementary Fig. 15). The enthalpy value is significantly lower than the reported value for covalently connected triptycene gears^15^,^19^.

It is noteworthy in connection with motion transmission that a certain number of Pt^II^ complexes display photo-driven *cis*/*trans* isomerization^30^,^31^. Under photoirradiation at 360 nm, *cis*-PtCl_2_**1**_2_ was found to be isomerized to *trans*-PtCl_2_**1**_2_ in C_6_H_6_ at room temperature (*cis*/*trans*=50:50 by ^1^H NMR in CDCl_3_ at 300 K). As a result of slow evaporation, *trans*-PtCl_2_**1**_2_ was isolated as yellow crystals in 29% yield (Supplementary Figs 16 and 17). In a ^31^P NMR spectrum of the *trans* isomer in CDCl_3_, a ^31^P-^195^Pt coupling (*J*_1_=2,937 Hz, in Supplementary Fig. 18) indicated that the two phosphine ligands bind to the central Pt^II^ ion. On the other hand, its ^1^H NMR spectrum in CDCl_3_ showed neither splitting nor upfield shift of the proton signals for the 4-positions, suggesting the absence of significant intramolecular interactions between the two rotators.

### X-ray crystallographic analyses

**Figure 3:**
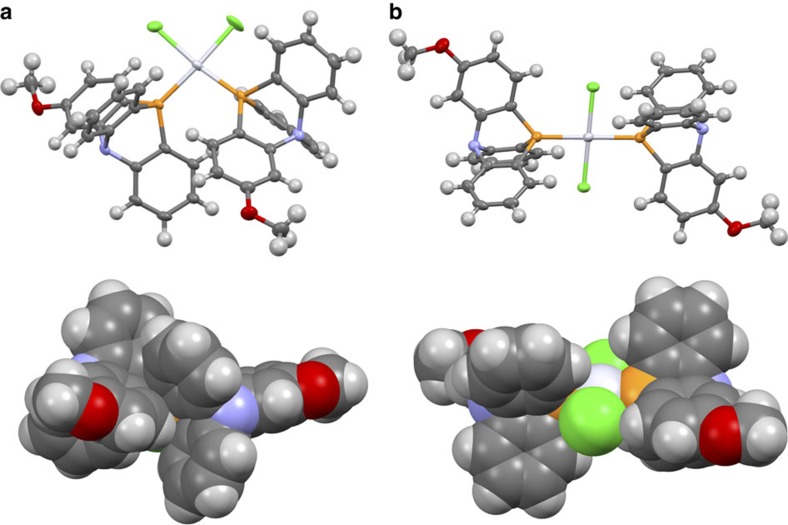
X-ray crystal structures of *cis*- and *trans*-PtCl_2_**1**_2_. (**a**) *cis*-PtCl_2_**1**_2_·(ether). (**b**) *trans*-PtCl_2_**1**_2_·(C_6_H_6_)_2_. In both cases, the structures are indicated as ORTEP (Oak Ridge Thermal Ellipsoid Plot) diagram with 50% thermal ellipsoid (upper) and space-filling model (bottom). Solvents are omitted for clarity, and colours are coded according to CPK (Corey, Pauling, Koltun) colouring.

Crystals of *cis*-PtCl_2_**1**_2_·(ether) suitable for single-crystal X-ray structure analysis were obtained by liquid–liquid diffusion of Et_2_O into a solution of PtCl_2_**1**_2_ in toluene. One molecule of diethyl ether was included into the unit structure (Fig. 3, Supplementary Fig. 19). The X-ray diffraction data demonstrated that the two rotators adopt an ‘engaged' *cis* form in the solid state. A unit cell consists of a *meso* isomer of PtCl_2_**1**_2_, one rotational isomer comes from tight meshing of the two rotators. Notably, intramolecular CH–π interactions were observed between the two rotators. Each Pt^II^ ion is in a distorted square planar geometry, in which the P–Pt–P angle is over 90° (99.95(5)°) due to the bulkiness of rotator **1**. The two rotators **1** are thus suitably engaged with each other on the Pt^II^ stator. In contrast, single-crystal X-ray structure analysis of *trans*-PtCl_2_**1**_2_·(C_6_H_6_)_2_ revealed that the two phosphine ligands as rotators are across from one another in the square planar Pt^II^ complex (Supplementary Fig. 20). This photoisomerized *trans* form can be regarded as a ‘disengaged' state of the metal-centred molecular gear.

### Photo- and thermally driven isomerization of PtCl_2_1_2_

**Figure 4:**
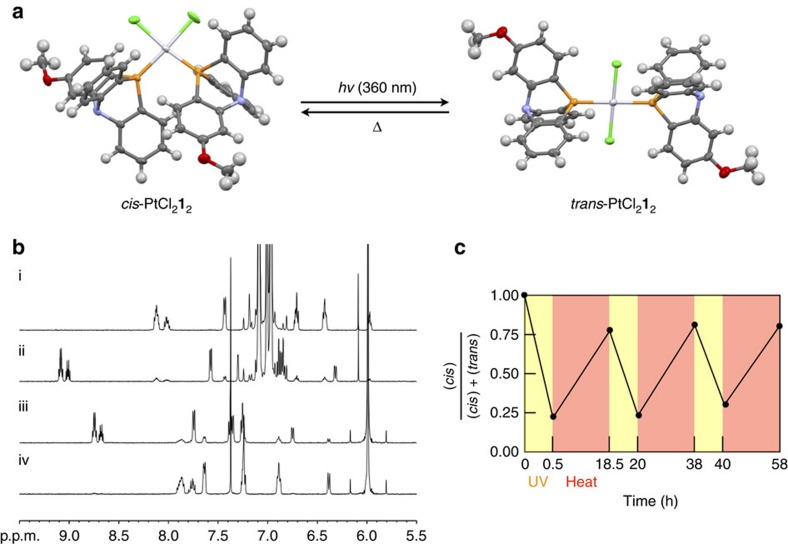
Photo and thermal switching of molecular gear PtCl_2_**1**_2_. (**a**) Photo- and thermally induced isomerization between *cis*- and *trans-*PtCl_2_**1**_2_. (**b**) ^1^H NMR spectra for photo- and thermally induced isomerization from *cis*- to *trans-*PtCl_2_**1**_2_ (500 MHz, 300 K). (i) A solution of single crystals of *cis*-PtCl_2_**1**_2_ in toluene-*d*_8_ (*cis*/*trans*=99:1); (ii) a solution of (i) after photoirradiation at 360 nm (after 30 min, *cis*/*trans*=15:85); (iii) a solution of single crystals of *trans*-PtCl_2_**1**_2_ in TCE-*d*_2_ (after 5 min, *cis*/*trans*=12:88); (iv) a solution of (iii) after 10 h at room temperature (*cis*/*trans*=2:98). (**c**) Reversible switching of the molecular gearing system, PtCl_2_**1**_2_, in TCE-*d*_2_/toluene-*d*_8_=1:1 (v v^−1^).

We then envisioned that this gear system could be applied to a stimuli-responsive molecular switch based on the photo- and thermally driven *cis*–*trans* isomerization in an appropriate solvent. It is well known that photo or thermal isomerization of diphosphine Pt^II^ complexes highly depends on the solvent polarity^8^. Polar solvents generally prefer *cis* form rather than *trans* form because the *cis* complex has a dipole moment that interacts better with the solvent polarity. Photo-driven isomerization from *cis* to *trans* form was then examined in a solvent with low polarity. Ultraviolet light at 360 nm was irradiated to a solution of pure *cis*-PtCl_2_**1**_2_ in toluene-*d*_8_ at room temperature (Fig. 4a,b). A photo stationary state was reached after 30 min, where the *cis*/*trans* ratio was changed to 15:85 (Supplementary Fig. 21). On the other hand, in more polar 1,1,2,2,-tetrachloroethane-*d*_2_ (TCE-*d*_2_), thermal isomerization from *trans* to *cis* was so fast in the dark at room temperature that it was difficult to obtain a ^1^H NMR spectrum of pure *trans* complex in the TCE-*d*_2_ solution because of the rapid isomerization to the *cis* form. After 10 h, the *trans* complex was transformed into *cis* form nearly quantitatively (*cis*/*trans*=98:2; Supplementary Fig. 22). This structural interconversion was repeatable in a mixed solvent of TCE-*d*_2_/toluene-*d*_8_=1:1. When a solution of pure *cis*-PtCl_2_**1**_2_ was irradiated by ultraviolet light at 360 nm, the *cis* to *trans* conversion proceeded smoothly at room temperature, and the reaction achieved its equilibrium (*cis*/*trans*=19:81) in 30 min. In this mixed solvent system, the interconversion from *trans* to *cis* was slow enough to determine the ratio of the complex by NMR at 300 K. When the *trans*-based solution was heated at 100 °C for 10 h, the *trans*-based solution was reversed to the *cis*-based solution with the *cis*/*trans*=78:22. This *cis*–*trans* isomerization process was thus repeatable at least three times by the repetition of stimuli (Fig. 4c and Supplementary Figs 23 and 24).

## Discussion

In conclusion, we have developed a molecular gear, PtCl_2_**1**_2_, composed of two azaphosphatriptycene rotators **1** with a Pt^II^ ion acting as a stator. The repeatable mechanical switching function based on the *cis*–*trans* isomerization at the Pt^II^ centre was achieved by photoirradiation and heating. Traceless external stimuli-responsive configurational changes of metal ions show promise as a movement element of molecular machines with a motion transmission function.

## Methods

### General information

Unless otherwise noted, solvents and reagents were purchased and used without further purification. 2-Methoxy-9-aza-10-phosphatriptycene (**1**) was synthesized from 3-anisidine (Supplementary Figs 1–10 and Supplementary Note 1).

^1^H, ^13^C, ^31^P NMR and other two-dimensional NMR spectra were recorded on a Bruker AVANCE III-500 (500 MHz) spectrometer. Tetramethylsilane was used as an internal standard (*δ* 0 p.p.m.) for ^1^H and ^13^C NMR measurements when CDCl_3_ was used as solvent. A residual solvent signal was used for calibration of ^1^H NMR measurements when other deuterated solvents (C_6_HD_5_: 7.16 p.p.m.; toluene-*d*_7_: 6.97 p.p.m.; 1,1,2,2-tetrachloroethane-*d*: 5.99 p.p.m.) was used as a solvent. ESI-TOF mass data were recorded on a Micromass LCT Premier XE mass spectrometer. Unless otherwise noted, experimental conditions were as follows: ion mode, positive; capillary voltage, 3,000 V; sample cone voltage, 30 V; desolvation temperature, 150 °C; source temperature, 80 °C). Melting point was measured by Yanaco Micro Melting Point Apparatus MP-500D and uncorrected. Elemental analysis was conducted in the Microanalytical Laboratory, Department Chemistry, Graduate School of Science, the University of Tokyo (Tokyo, Japan). Infrared spectra were recorded on a Jasco FT/IR 4,200 with an ATR equipment.

### Synthesis of *cis*-PtCl_2_ **1**_2_

To a 5.0 mM solution of K_2_PtCl_4_/H_2_O (40 ml, 0.20 mmol, 1.0 eq) was added a 10 mM solution of 2-methoxy-9-aza-10-phosphatriptycene (**1**) in EtOH (40 ml, 0.40 mmol, 2.0 eq). The suspended solution was then stirred at room temperature for 21 h in the dark. The resulting precipitate was collected by filtration, washed with H_2_O and EtOH, and dried under vacuum to give a colourless solid (144 mg). The crude product was purified by recrystallization from chloroform/diethyl ether to give *cis*-PtCl_2_**1**_2_ (112 mg, 0.118 mmol, 59%) as a colourless solid. ^1^H NMR (C_6_D_6_, 500 MHz, 300 K): *δ* 7.81–7.78 (m, 4H), 7.71–7.66 (m, 2H), 7.05 (d, *J*=7.6 Hz, 4H), 6.83 (s, 2H), 6.31–6.27 (m, 4H), 6.04–6.00 (m, 4H), 5.62–5.68 (m, 2H), 2.57 (s, 2H), 2.55 (s, 4H); ^31^P NMR (C_6_D_6_, 202 MHz, 300 K): *δ* −31.0 (*J*_P–Pt_=3,625 Hz); HRMS (CHCl_3_/CH_3_CN/HCO_2_H, positive): [PtCl**1**_2_]^+^ (C_38_H_28_ClN_2_O_2_P_2_Pt) *m*/*z* 836.0958 (required, 836.0957).

### Synthesis of *trans*-PtCl_2_1_2_

In a 50 ml three-necked flask, a solution of *cis*-PtCl_2_**1**_2_ (20.0 mg, 21 μmol) in benzene was irradiated at 360 nm for 1 h at room temperature. The solvent was removed by evaporation to give a yellow solid (22.5 mg). The crude product was recrystallized from benzene (2 ml) to obtain yellow crystals of *trans*-PtCl_2_**1**_2_·(C_6_H_6_)_2_. After dryness, 5.8 mg of desired complex was obtained, which contains 0.5 eq of benzene (confirmed by NMR, 6.1 μmol, 29%). ^1^H NMR (CDCl_3_, 500 MHz, 300 K): *δ* 8.78–8.75 (m, 4H), 8.70 (dt, *J*=8.4, 5.4 Hz, 2H), 7.66 (dd, *J*=7.7, 1.1 Hz, 4H), 7.34 (td, *J*=7.6, 1.2 Hz, 4H), 7.28–7.25 (m, 2H), 7.22 (td, *J*=7.5, 1.3 Hz, 4H), 6.72 (dt, *J*=8.4, 1.2 Hz, 2H), 3.82 (s, 6H); ^31^P NMR (CDCl_3_, 202 MHz, 300 K): *δ* −39.0 (*J*_P–Pt_=2,937 Hz); HRMS (CHCl_3_/CH_3_CN/HCO_2_H, positive): [PtCl**1**_2_]^+^ (C_38_H_28_ClN_2_O_2_P_2_Pt) *m*/*z* 836.0958 (required, 836.0957).

### Photo- and thermally driven isomerization of PtCl_2_ **1**_2_

A 1.0 mM solution of *cis*-PtCl_2_**1**_2_ in TCE-*d*_2_/toluene-*d*_8_=1:1 (600 μl, 0.60 μmol) and a 100 mM solution of 1,4-dioxane in TCE-*d*_2_/toluene-*d*_8_=1:1 (6.0 μl, 0.60 μmol) were placed in an NMR tube, which was sealed by a septum rubber and degassed by freeze–pump–thaw three times. The reaction mixture was irradiated with ultraviolet lamp (ASAHI, MAX-303) using 360 nm filter (bandwidth=10 nm) at room temperature, and was heated at 100 °C in the dark (Supplementary Figs 19–22).

### X-ray diffraction analysis

Single-crystal X-ray crystallographic analyses were performed using a Rigaku Saturn724+ diffractometer with MoK*α* radiation (for *cis*-PtCl_2_**1**_2_) or Rigaku RAXIS-RAPID imaging plate diffractometer with MoK*α* radiation (for *trans*-PtCl_2_**l**_2_), and obtained data were calculated using the Crystal Structure crystallographic software package except for refinement, which was performed using SHELXL-2014 (ref. 33). All hydrogen atoms were placed geometrically and refined using a riding model. Details for the synthesis and X-ray diffraction mesurements of both *cis*- and *trans*-complex are given in CIF files and Supplementary Figs 19 and 20 and Supplementary Note 2.

### Data availability

Crystallographic data in this paper can be obtained free of charge from the Cambridge Crystallographic Data Centre (http://www.ccdc.cam.ac.uk/data_request/cif). The Deposit numbers are 1404948 (*cis*-PtCl_2_**1**_2_) and 1404949 (*trans*-PtCl_2_**1**_2_), respectively. All other data are available on this article and its Supplementary Information file.

## Additional information

**How to cite this article:** Ube, H. *et al*. Metal-centred azaphosphatriptycene gear with a photo- and thermally driven mechanical switching function based on coordination isomerism. *Nat. Commun.* **8,** 14296 doi: 10.1038/ncomms14296 (2017).

**Publisher's note**: Springer Nature remains neutral with regard to jurisdictional claims in published maps and institutional affiliations.

## Supplementary Material

Supplementary Information Supplementary Figures, Supplementary Notes, Supplementary Methods and Supplementary References.

Peer review file
