# Supplementary material for: Metal-centred azaphosphatriptycene gear with a photo- and thermally driven mechanical switching function based on coordination isomerism
Source: Nat Commun. 2017 Feb 8;8:14296. doi: 10.1038/ncomms14296 (PMC5309778; doi:10.1038/ncomms14296)
Supplement: Supplementary Information — Supplementary Figures, Supplementary Notes, Supplementary Methods and Supplementary References. [file ncomms14296-s1.pdf]

Supplementary Figures

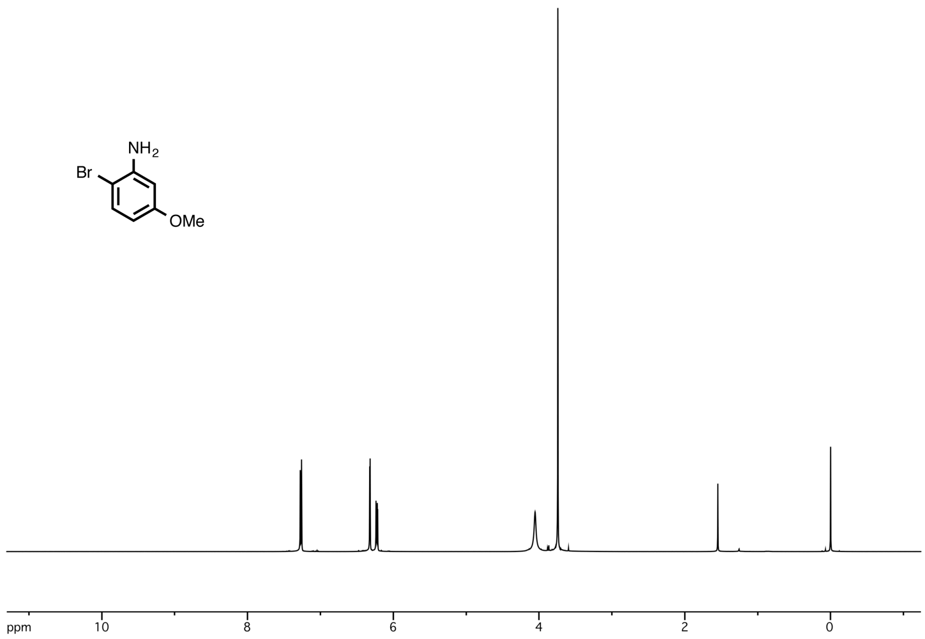

Supplementary Figure 1. ^{1}H NMR spectrum of **2** ($CDCl_3$, 500 MHz, 300 K).

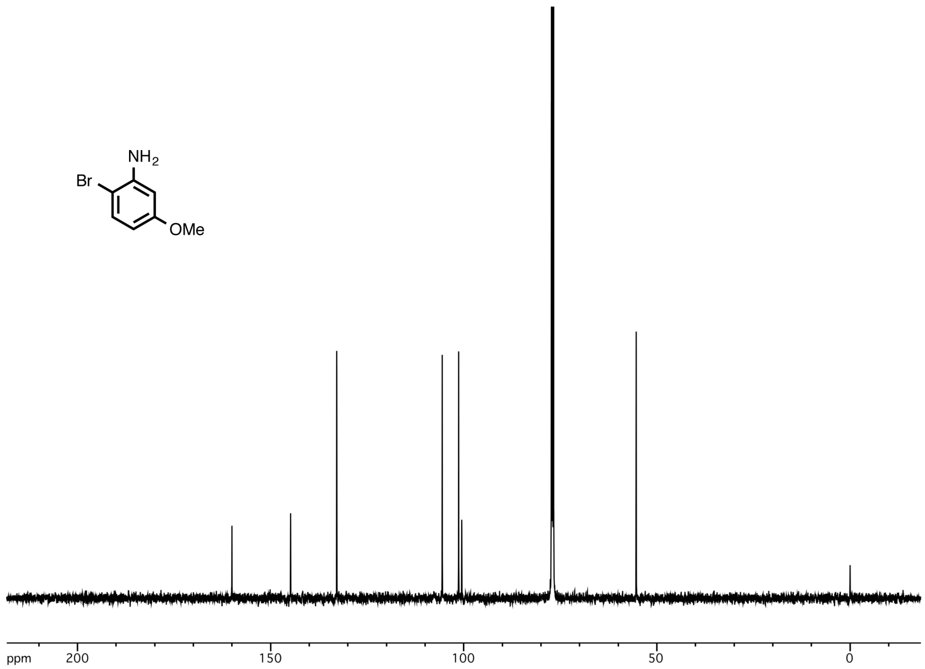

Supplementary Figure 2. ^{13}C NMR spectrum of **2** ($CDCl_3$, 126 MHz, 300 K).

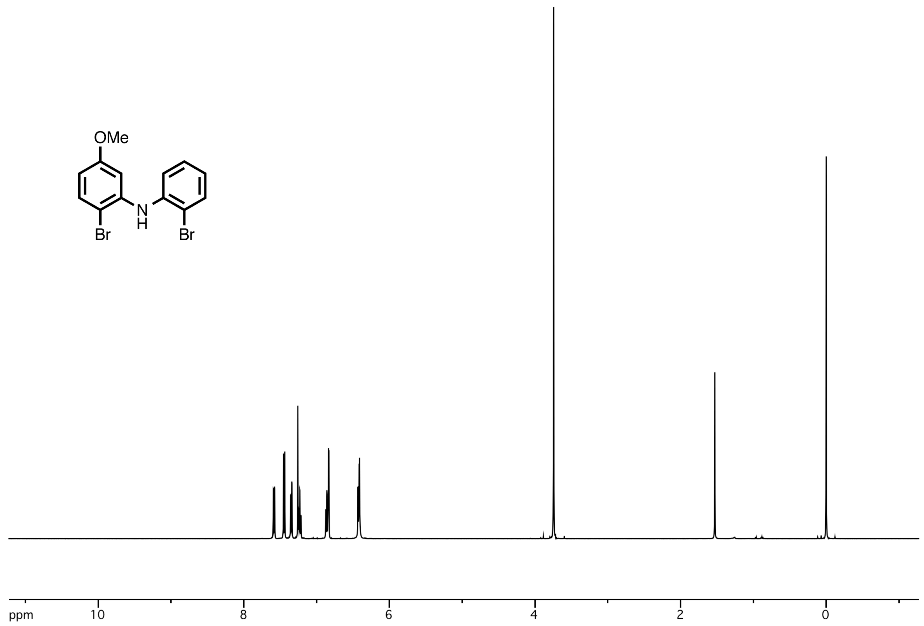

Supplementary Figure 3. ^{1}H NMR spectrum of **3** ($CDCl_3$, 500 MHz, 300 K).

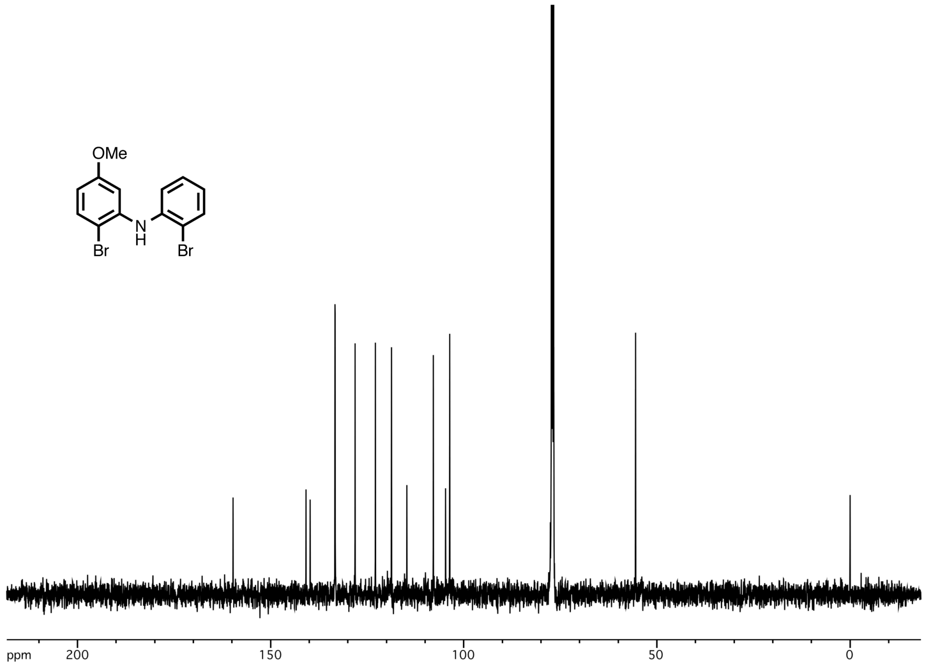

Supplementary Figure 4. ^{13}C NMR spectrum of **3** ($CDCl_3$, 126 MHz, 300 K).

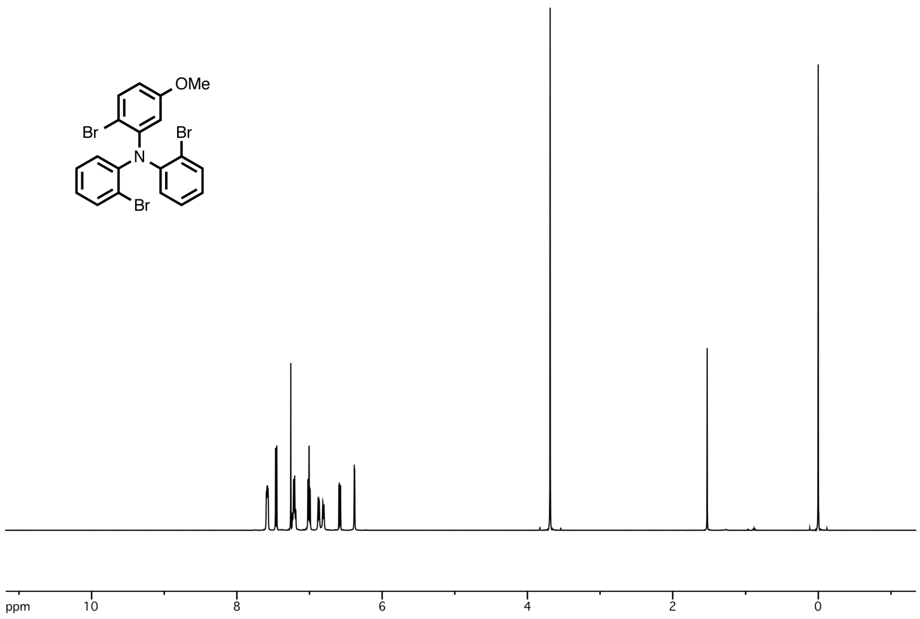

Supplementary Figure 5. ^{1}H NMR spectrum of **4** ($CDCl_3$, 500 MHz, 300 K).

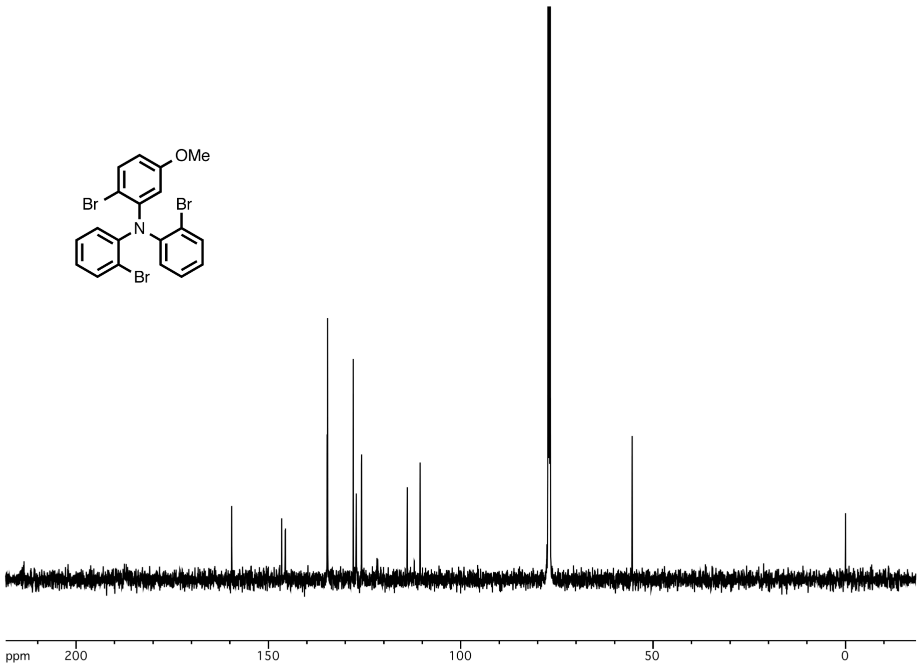

Supplementary Figure 6. ^{13}C NMR spectrum of **4** ($CDCl_3$, 126 MHz, 300 K).

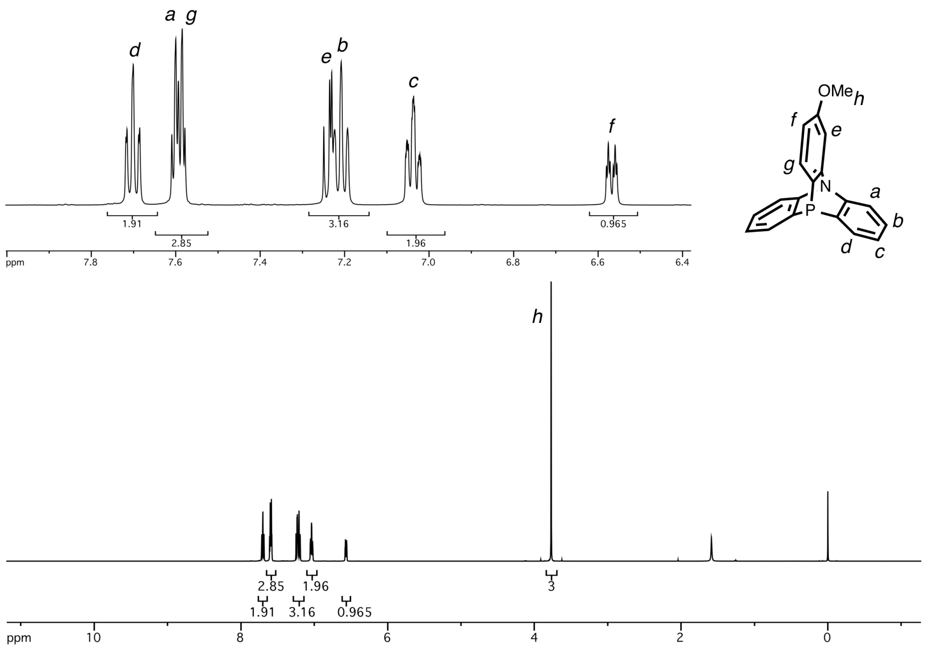

Supplementary Figure 7. ^{1}H NMR spectrum of **1** ($CDCl_3$, 500 MHz, 300 K).

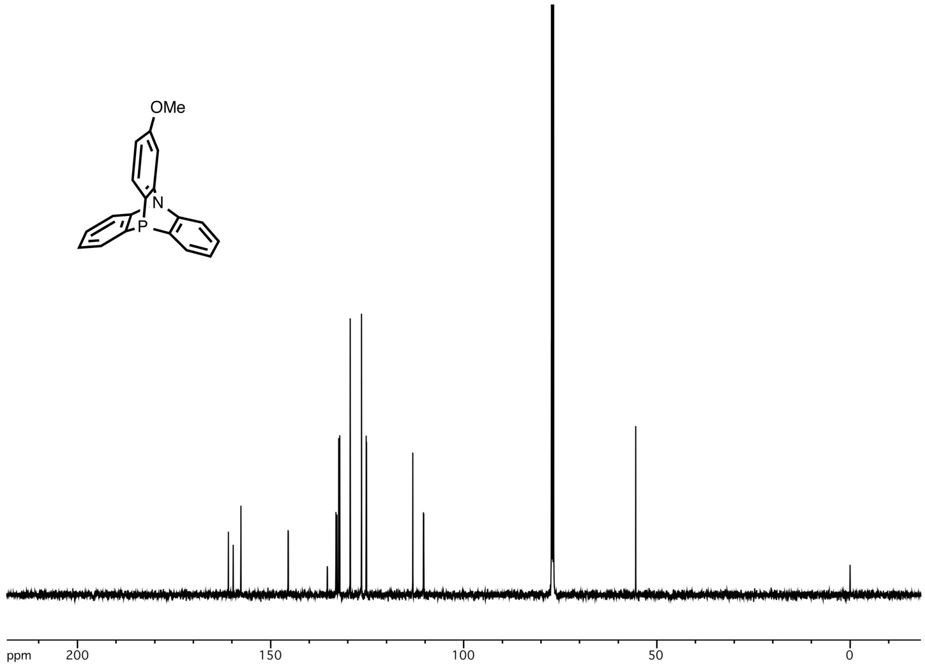

Supplementary Figure 8. ^{13}C NMR spectrum of **1** ($CDCl_3$, 126 MHz, 300 K).

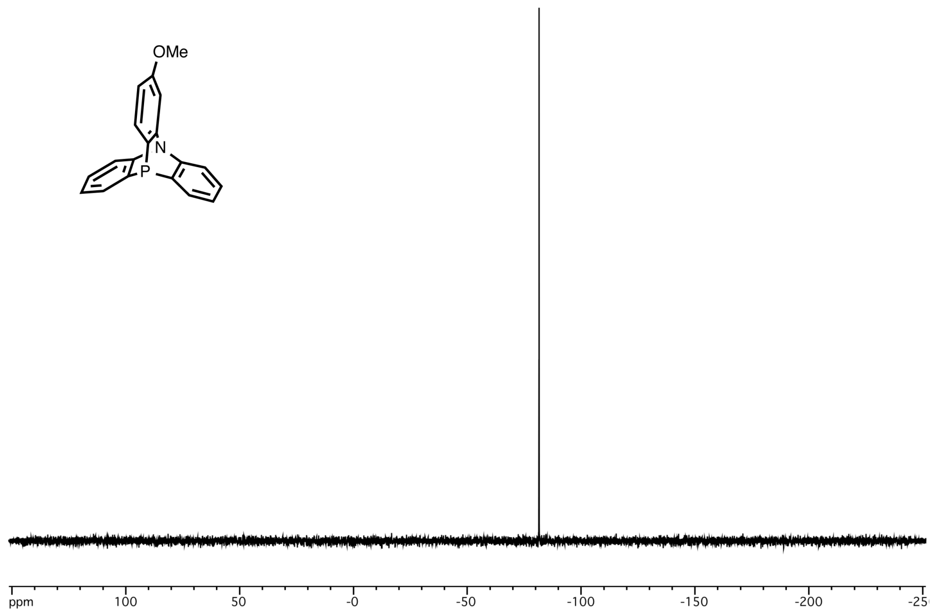

Supplementary Figure 9. ^{31}P NMR spectrum of **1** ($CDCl_3$, 202 MHz, 300 K).

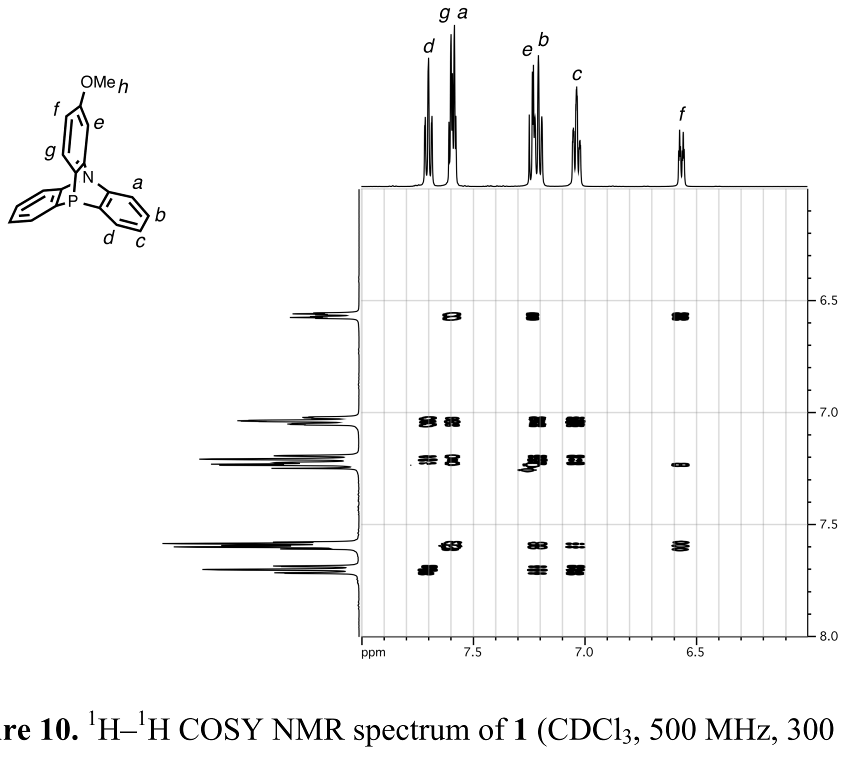

Supplementary Figure 10. ^{1}H–^{1}H COSY NMR spectrum of **1** ($CDCl_3$, 500 MHz, 300 K).

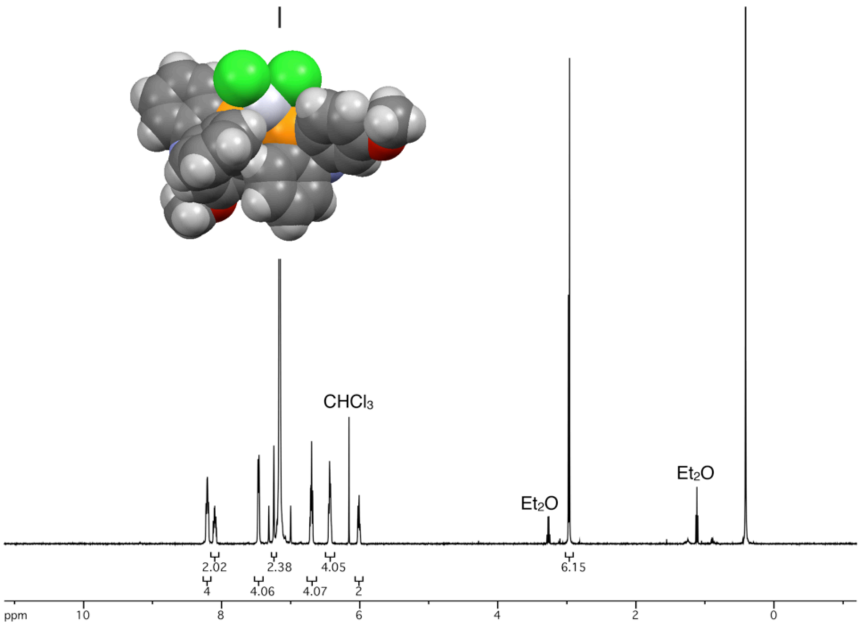

Supplementary Figure 11. ^{1}H NMR spectrum of *cis*-PtCl$_2$**1**$_2$ (C_6D_6, 500 MHz, 300 K).

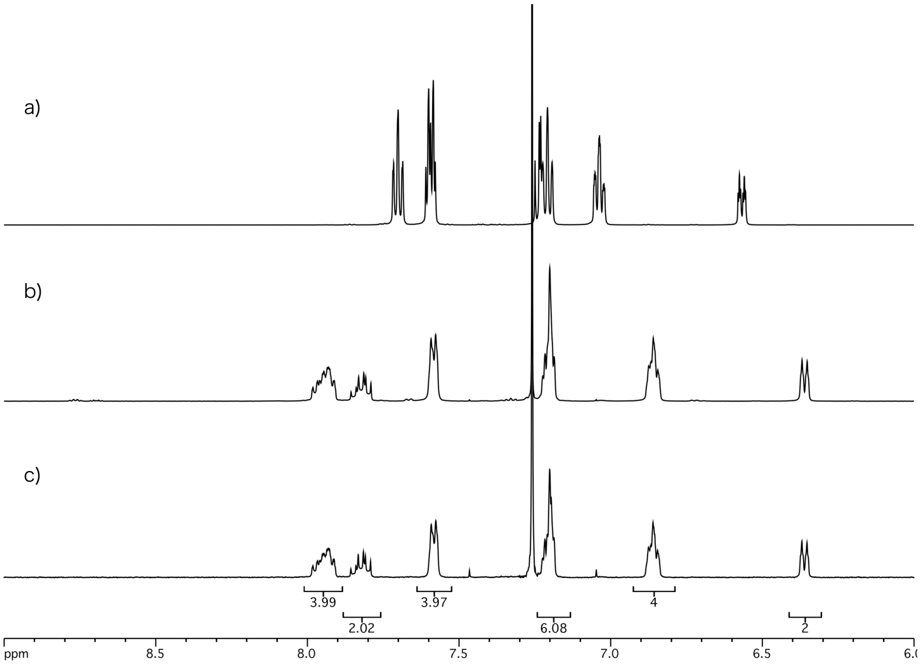

Supplementary Figure 12.^{1}H NMR spectra of *cis*-PtCl$_2$**1**$_2$ ($CDCl_3$, 500 MHz, 300 K). a) **1**, b) crude product and c) *cis*-PtCl$_2$**1**$_2$ after reprecipitation from $CHCl_3$/diethyl ether.

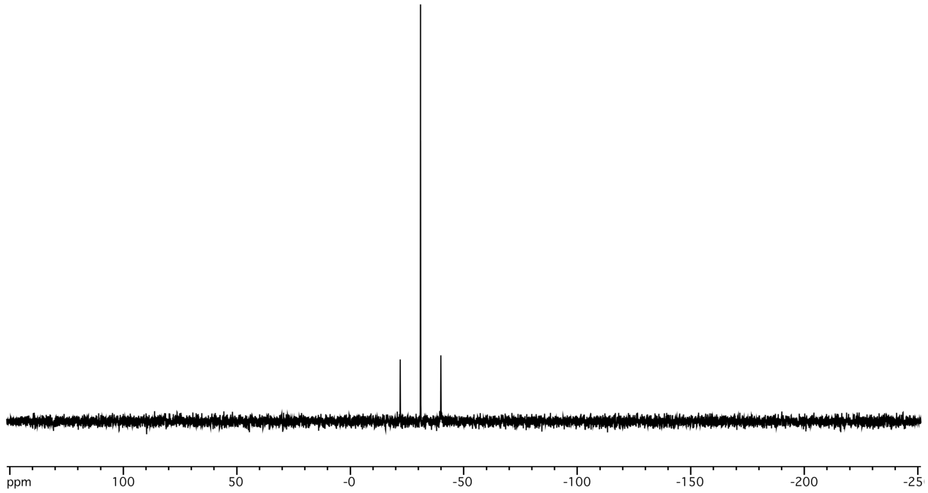

Supplementary Figure 13. ^{31}P NMR spectrum of *cis*-$PtCl_2$**1**$_2$ (C_6D_6, 202 MHz, 300 K).

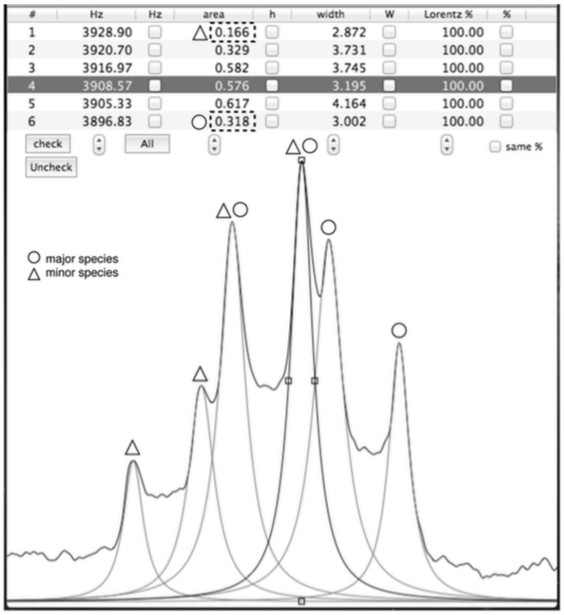

Supplementary Figure 14. Line shape analysis of *cis*-$PtCl_2$**1**$_2$ (C_6D_6, 500 MHz, 300 K). A 2:1 mixture of *dl* rotational isomers was well separated.

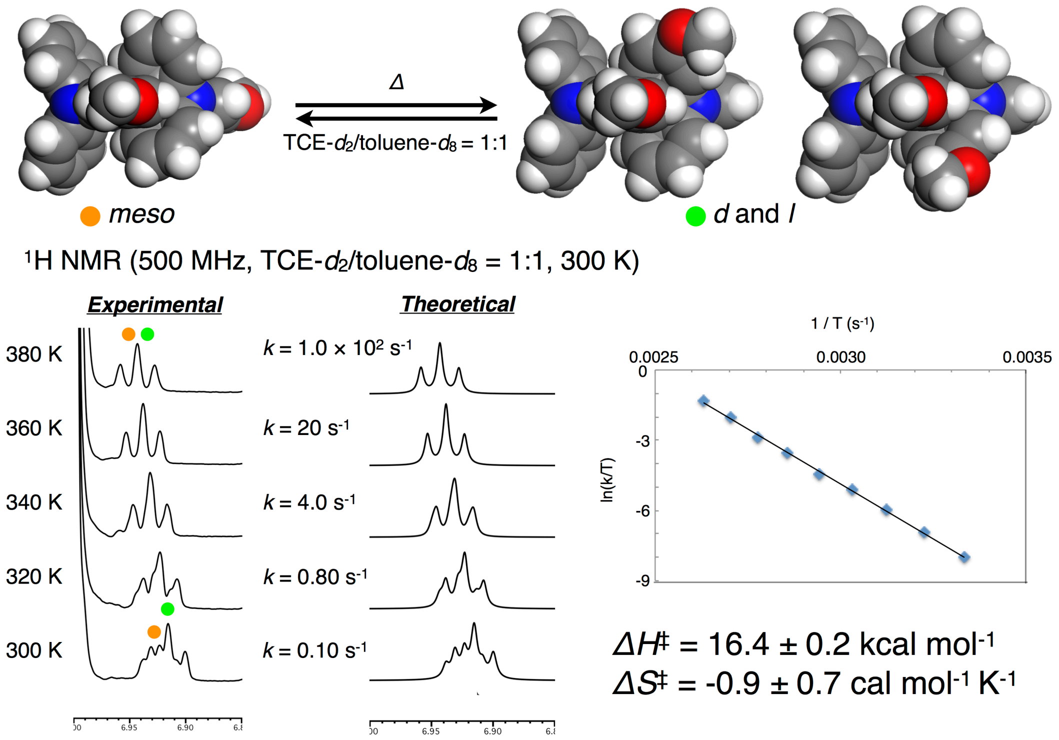

Supplementary Figure 15. Quantitative analysis of gear slippage parameters of *cis*-$PtCl_2\mathbf{1}_2$. The activation parameters for the gear slippage process was determined by interconversion between *meso* and *dl cis*-$PtCl_2\mathbf{1}_2$. Signals of the 7-position protons of azaphosphatriptycenes were simulated by the two-site exchange model from 300 K to 380 K. Parameters were estimated by Eyring plot.

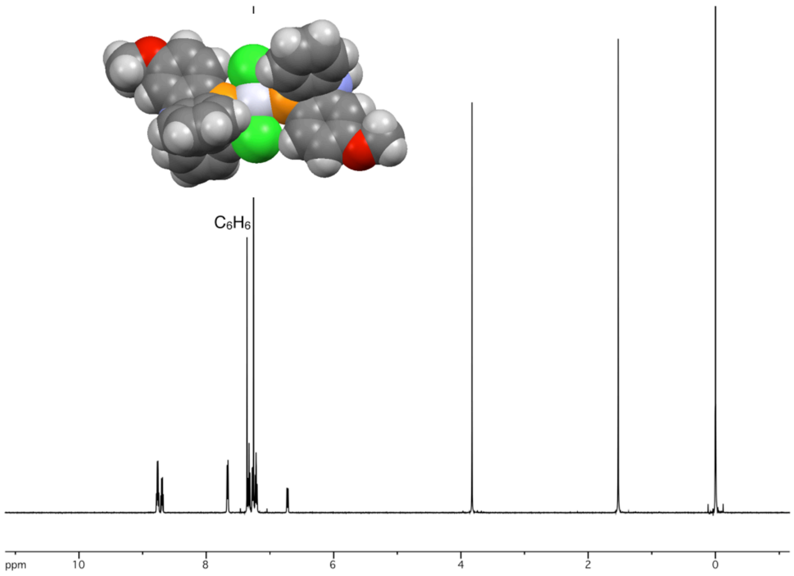

Supplementary Figure 16. ^{1}H NMR spectrum of *trans*-PtCl$_2$**1**$_2$ (CDCl$_3$, 500 MHz, 300 K).

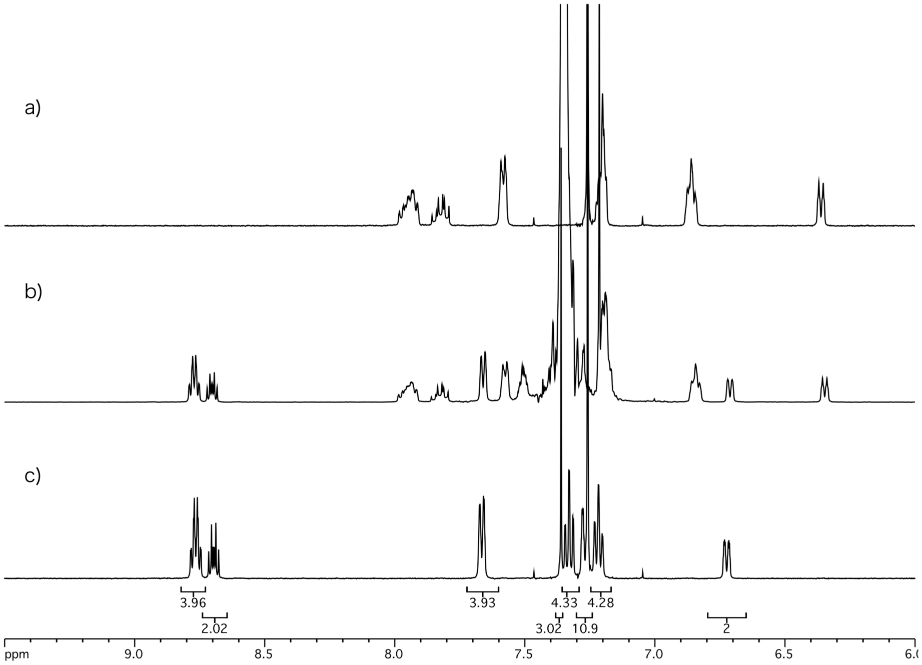

Supplementary Figure 17. ^{1}H NMR spectra of *trans*-PtCl$_2$**1**$_2$ (CDCl$_3$, 500 MHz, 300 K). a) *cis*-PtCl$_2$**1**$_2$, b) crude product and c) *trans*-PtCl$_2$**1**$_2$ after recrystallization from benzene.

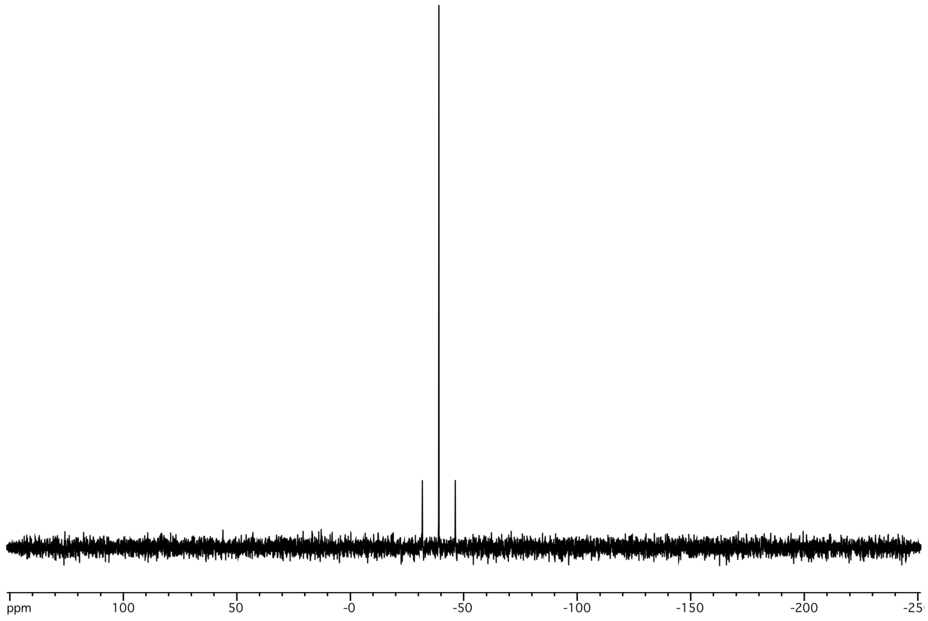

Supplementary Figure 18. ^{31}P NMR spectrum of *trans*-$PtCl_2$**1**$_2$ ($CDCl_3$, 202 MHz, 300 K).

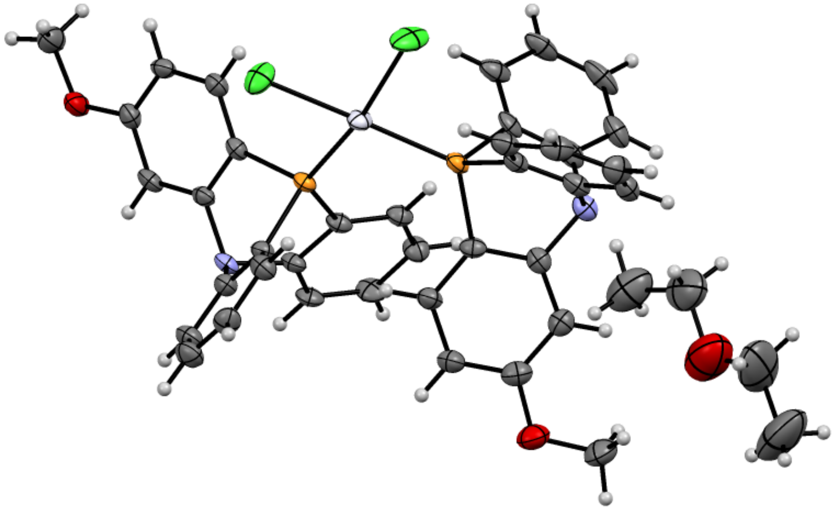

Supplementary Figure 19. Crystal structure of *cis*-$PtCl_2\mathbf{1}_2$. Thermal ellipsoids set at 50% probability. Color code; C gray, N blue, O red, P orange, Cl green, Pt white.

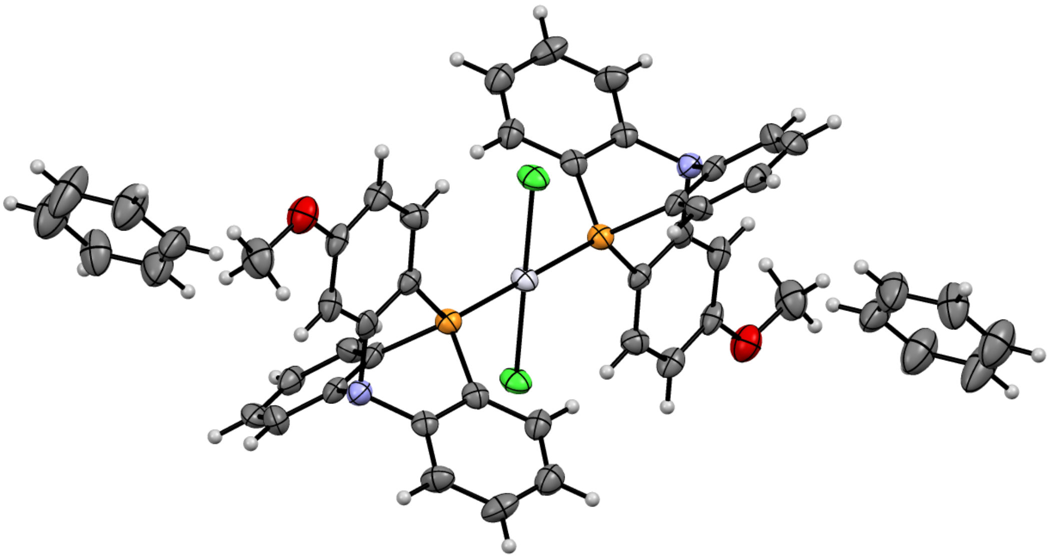

Supplementary Figure 20. Crystal structure of *trans*-$PtCl_2\mathbf{1}_2\cdot(C_6H_6)_2$. Thermal ellipsoids set at 50% probability. Color code; C gray, N blue, O red, P orange, Cl green, Pt white.

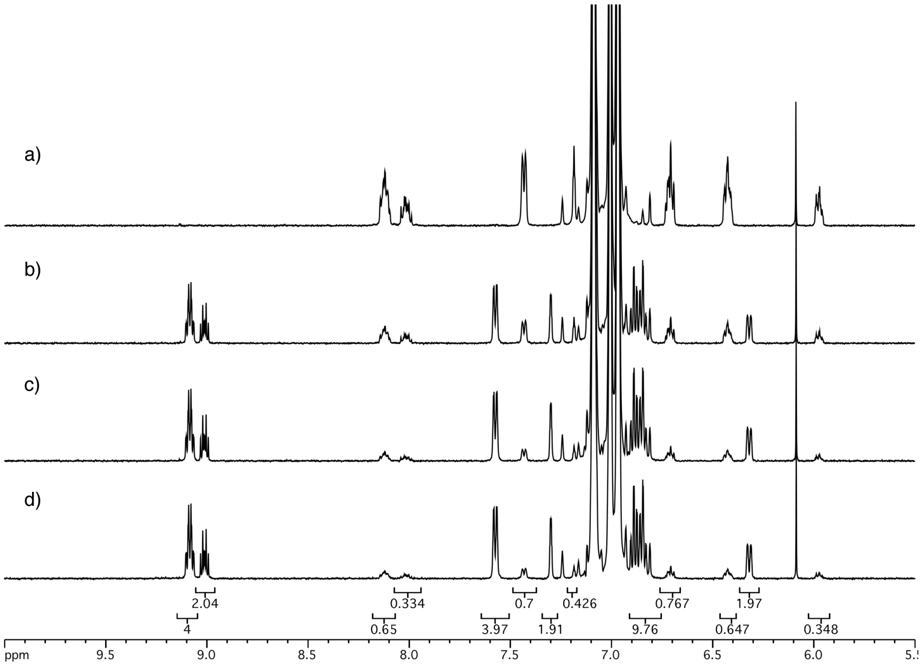

Supplementary Figure 21. ^{1}H NMR study of photo-induced *cis* to *trans* isomerization of $PtCl_2\mathbf{1}_2$ (toluene-d_8, 500 MHz, 300 K). a) *cis*-$PtCl_2\mathbf{1}_2$, b), c) and d) a) + UV irradiation at room temperature for 10 min (b), 20 min (c) and 30 min (d).

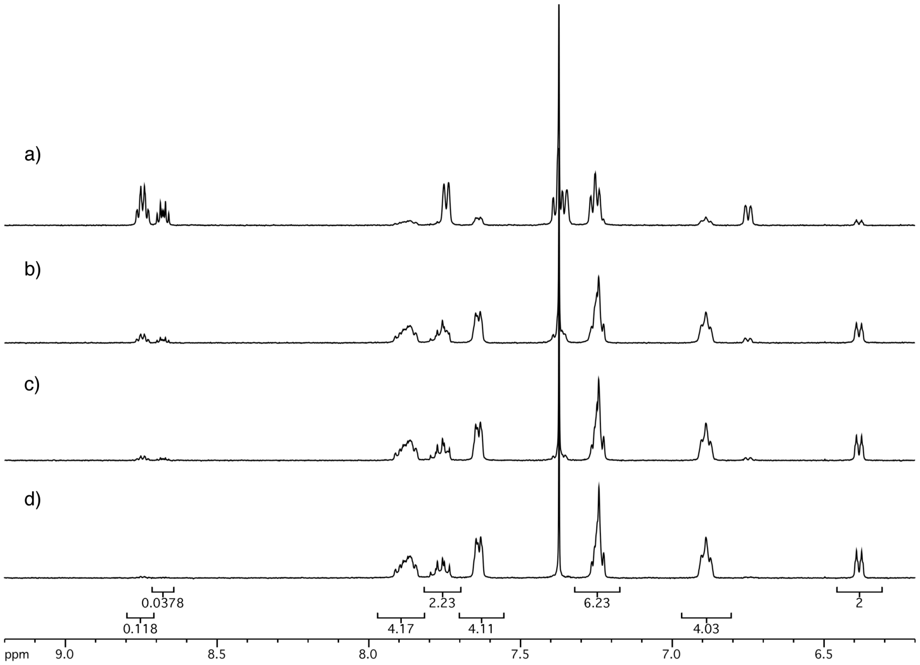

Supplementary Figure 22. ^{1}H NMR study of thermally-induced *trans* to *cis* isomerization of $PtCl_2\mathbf{1}_2$ (TCE-d_2, 500 MHz, 300 K). a) *trans*-$PtCl_2\mathbf{1}_2$ after 5 min, b) 1 h, c) 2h and d) 10 h.

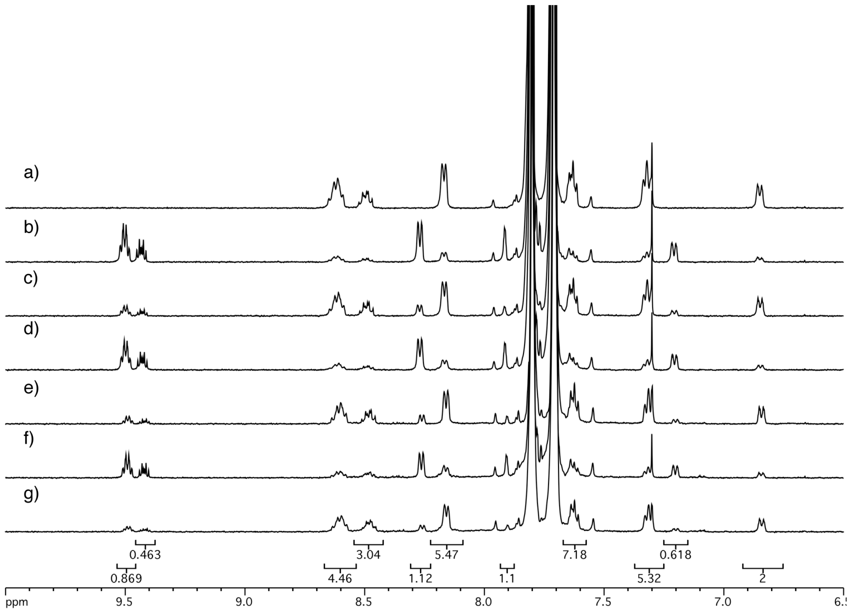

Supplementary Figure 23. ^{1}H NMR study of photo- and thermally-induced *cis*-*trans* isomerization of $PtCl_2\mathbf{1}_2$ (TCE-d_2/toluene-d_8 = 1:1 (v/v %), 500 MHz, 300 K). a) *cis*-$PtCl_2\mathbf{1}_2$, b) a) + UV irradiation at room temperature for 0.5 h, c) b) + heating at 100 °C for 18 h, d) c) + UV irradiation at room temperature for 1.5 h, e) d) + heating at 100 °C for 18 h, f) e) + UV irradiation at room temperature for 2 h and g) f) + heating at 100 °C for 18 h.

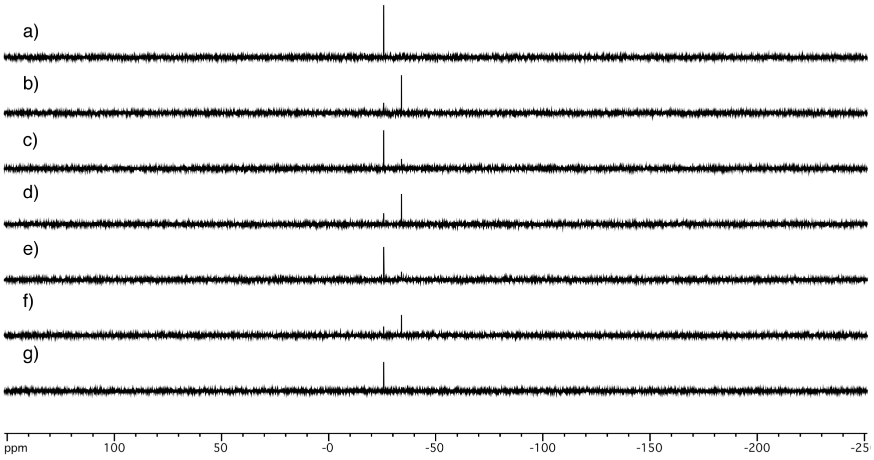

Supplementary Figure 24. ^{31}P NMR study of photo- and thermally-induced *cis*-*trans* isomerization of $PtCl_2\mathbf{1}_2$ (TCE-d_2/toluene-d_8 = 1:1 (v/v%), 202 MHz, 300 K). a) *cis*-$PtCl_2\mathbf{1}_2$, b) a) + UV irradiation at room temperature for 0.5 h, c) b) + heating at 100 °C for 18 h, d) c) + UV irradiation at room temperature for 1.5 h, e) d) + heating at 100 °C for 18 h f) e) + UV irradiation at room temperature for 2 h and g) f) + heating at 100 °C for 18 h.

Supplementary Note 1

Synthesis of 2-bromo-5-methoxyaniline (2) [1]

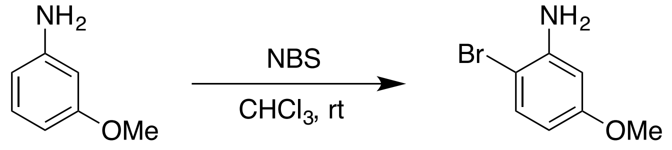

2

A three-necked 100 mL flask equipped with a magnetic stirring bar was filled with Ar gas. 3-Anisidine (5.60 mL, 50.0 mmol, 1.0 eq) was dissolved in dry $CHCl_3$ (50 mL) at 6 °C, to which was added *N*-bromosuccinimide (8.90 g, 50.0 mmol, 1.0 eq) portionwise. The reaction mixture was warmed to room temperature and stirred for 11 h, and then quenched by saturated $Na_2S_2O_3$ aqueous solution. The organic layer was separated and washed with 1.0 M NaOH aqueous solution (50 mL × 2), H_2O (50 mL) and brine (50 mL), and dried over anhydrous $MgSO_4$. After filtration and evaporation, the obtained brown liquid (11.3 g) was purified by silica gel column chromatography (Merck 230-400 mesh, ϕ = 80 mm, H = 15 cm, 1st: *n*-hexane/CH_2Cl_2 = 2:1, 2nd: *n*-hexane/AcOEt = 12:1) to obtain **2** as a brown liquid (5.80 g, 28.7 mmol, 57%).

^{1}H NMR ($CDCl_3$, 500 MHz, 300 K): δ 7.27 (d, J = 8.8 Hz, 1H), 6.32 (d, J = 2.8 Hz, 1H), 6.23 (dd, J = 8.8, 2.8 Hz, 1H), 4.05 (s, 2H), 3.74 (s, 3H); ^{13}C NMR ($CDCl_3$, 126 MHz, 300 K): δ 160.0, 144.8, 132.9, 105.6, 101.3, 100.5, 55.4; FT-IR: 3465.5, 3371.0, 3200.3 (N-H stretch), 3001.7 (aromatic C-H stretch), 2960.2, 2938.0 (methyl C-H stretch), 1612.2 (N-H bend), 1575.6, 1488.8 (aromatic C=C stretch), 825.4 (aromatic C-H bend (2)) cm^{-1}; MS ($CHCl_3/CH_3CN/HCO_2H$, positive): $[\mathbf{2}\cdot H]^+$ (C_7H_9BrNO) m/z 201.99 (required, 201.98).

Synthesis of 2-bromo-*N*-(2-bromophenyl)-5-methoxyaniline (3)

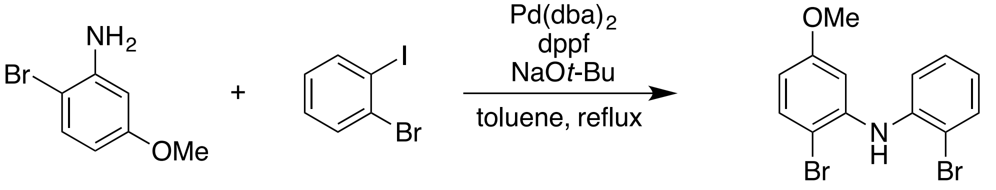

2 **3**

A three-necked flask equipped with a reflux condenser and a magnetic stirring bar was filled with Ar gas. To the flask were added 2-bromo-5-methoxyaniline (**2**) (3.30 mL, 25.0 mmol, 1.0 eq), 2-bromoiodobenzene (3.21 mL, 7.07 g, 25.0 mmol, 1.0 eq), bis(dibenzylideneacetone)palladium(0) (720 mg, 1.25 mmol, 0.050 eq), 1,1'-bis(diphenylphosphino)ferrocene (692 mg, 1.25 mmol, 0.050 eq), sodium *tert*-butoxide (3.60 g, 37.5 mmol, 1.5 eq) and dry toluene (50 mL), and degassed and filled with Ar. The reaction mixture was heated at reflux for 8 h. After cooling, the mixture was filtered through a celite pad with suction and the filtrate was evaporated to give a dark brown liquid. The residue was

diluted with AcOEt (100 mL) and the organic layer was washed with H_2O (50 mL × 2) and brine (50 mL), and then dried over anhydrous $MgSO_4$. After filtration, the solvent was removed under reduced pressure. The obtained brown residue (10.4 g) was purified by silica gel column chromatography (Merck 230-400 mesh, ϕ = 80 mm, H = 12 cm, *n*-hexane) to obtain **3** as a colorless solid (7.76 g, 21.7 mmol, 87%).

^{1}H NMR ($CDCl_3$, 500 MHz, 300 K): δ 7.58 (dd, J = 8.0, 1.3 Hz, 1H), 7.44 (d, J = 8.8 Hz, 1H), 7.35 (dd, J = 8.1, 1.3 Hz, 1H), 7.25-7.21 (m, 1H), 6.87-6.83 (m, 2H), 6.43-6.41 (m, 2H), 3.74 (s, 3H); ^{13}C NMR ($CDCl_3$, 126 MHz, 300 K): δ 159.7, 140.8, 139.8, 133.33, 133.29, 128.1, 122.9, 118.7, 114.7, 107.9, 104.7, 103.6, 55.5; mp: 68.8-69.7 °C; FT-IR: 3382.5 (N-H stretch), 3062.4 (aromatic C-H stretch), 2919.7 (methyl C-H stretch), 1586.2 (N-H bend), 1519.6, 1475.3 (aromatic C=C stretch), 821.5 (aromatic C-H bend (2)), 736.7 (aromatic C-H bend (4)) cm^{-1}; HRMS ($CHCl_3/CH_3CN/HCO_2H$, positive): [**3**·H]$^+$ ($C_{13}H_{12}Br_2NO$) *m/z* 355.9229 (required, 355.9280).

Synthesis of 2-bromo-*N*,*N*-bis(2-bromophenyl)-5-methoxyaniline (4)

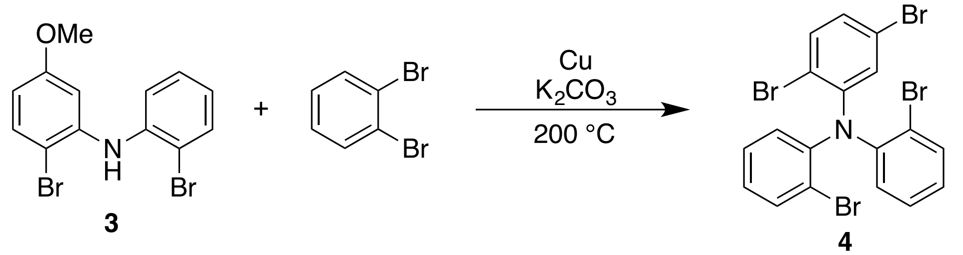

A three-necked flask equipped with a reflux condenser and a magnetic stirring bar was filled with Ar gas. Compound **3** (7.14 g, 20.0 mmol, 1.0 eq) and 1,2-dibromobenzene (4.84 mL, 40.0 mmol, 2.0 eq), copper powder (1.27 g, 20.0 mmol, 1.0 eq) and potassium carbonate (5.53 g, 40.0 mmol, 2.0 eq) were placed in the flask, and the reaction mixture was then heated in a 200 °C mantle heater for 53 h in total. After cooling, copper powder (1.27 g, 20.0 mmol, 1.0 eq) and potassium carbonate (5.53 g, 40.0 mmol) were further added to the mixture after 12 h. After cooling, a mixture of CH_2Cl_2 (200 mL) and H_2O (200 mL) was added to the reaction mixture containing generated brown solid, which was filtered through a pad of celite. The organic layer was separated and the aqueous layer was extracted with CH_2Cl_2 (100 mL × 2). The organic layers were combined, washed with 10% ammonia aqueous solution (100 mL × 2), H_2O (100 mL) and brine (100 mL), and then dried over anhydrous $MgSO_4$, filtrated and evaporated. The obtained brown liquid (16.1 g) was purified by silica gel column chromatography (Merck 230-400 mesh, ϕ = 80 mm, H = 14 cm, *n*-hexane/CH_2Cl_2 = 1:0 ~ 8:1) to obtain **4** as a colorless solid (4.81 g, 9.39 mmol, 47%).

^{1}H NMR ($CDCl_3$, 500 MHz, 300 K): δ 7.58 (m, 2H), 7.46 (d, J = 8.7 Hz, 1H), 7.22 (q, J = 7.2 Hz, 2H), 7.01 (td, J = 7.7, 1.3 Hz, 2H), 6.87 (d, J = 7.8 Hz, 1H), 6.82 (d, J = 7.8 Hz, 1H), 6.58 (dd, J = 8.8, 2.9 Hz, 1H), 6.38 (d, J = 2.9 Hz, 1H), 3.67 (s, 3H); ^{13}C NMR ($CDCl_3$, 126 MHz, 300 K): δ 159.5, 146.5,

145.65, 145.56, 134.7, 134.6, 128.0, 127.2, 125.82, 125.76, 113.9, 110.6, 55.4; mp: 147.7-148.5 °C; FT-IR: 3001.7 (aromatic C-H stretch), 2928.4 (methyl C-H stretch), 1596.8, 1574.6 (aromatic C=C bend), 791.7 (aromatic C-H bend (2), 757.9 (aromatic C-H bend (4)) cm^{-1}; HRMS ($CHCl_3/CH_3CN/HCO_2H$, positive): $[\mathbf{4}\cdot H]^+$ ($C_{19}H_{15}Br_3NO$) *m/z* 509.8672 (required, 509.8698).

Synthesis of 2-methoxy-9-aza-10-azaphosphatriptycene (1)

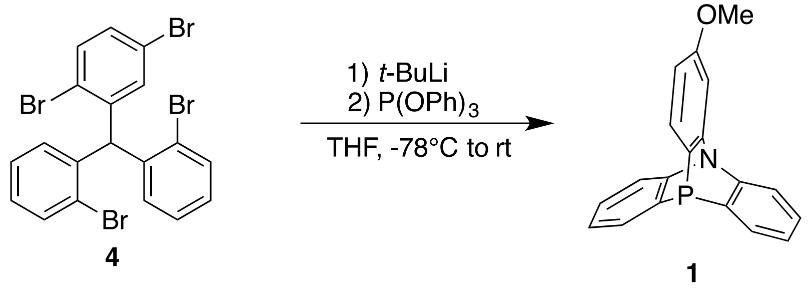

A three-necked 200 mL flask equipped with a magnetic stirring bar was filled with Ar gas. Anhydrous tetrahydrofuran (50 mL) was poured into the flask and stirred at -78 °C, to which was added *t*-BuLi in a pentane solution (1.77 M, 17.8 mL, 31.5 mmol, 6.3 eq). 2-Bromo-*N*,*N*-bis(2-bromophenyl)-5-methoxybenzenamine (**4**; 2.56 g, 5.00 mmol, 1.0 eq) dissolved in dry THF (50 mL) was then added dropwise to the solution. The reaction mixture was stirred at -78 °C for 90 min (‘solution A’). Separately, a three-necked 200 mL flask equipped with a magnetic stirring bar was filled with Ar gas. To the flask were added triphenyl phosphite (1.31 mL, 1.55 g, 5.00 mmol, 1.0 eq) and dry tetrahydrofuran (100 mL) (‘solution B). Then, a three-necked 500 mL flask equipped with two dropping funnels and a magnetic stirring bar was filled with Ar gas, to which anhydrous tetrahydrofuran (100 mL) was added. To the stirred solution were added dropwise both the ‘solution A” and ‘solution B’ in this order over 1 h using the dropping funnels at room temperature. The reaction mixture was further stirred at room temperature for 1 h. The reaction mixture was quenched with saturated NH_4Cl aqueous solution and the organic layer was separated and the solvent was evaporated. The residue was diluted with H_2O (100 mL), which was extracted with 100 mL of $CHCl_3$ three times. The organic layers were combined, washed with 1.0 M NaOH aqueous solution (50 mL × 2), H_2O (50 mL) and brine (50 mL), dried over $MgSO_4$, filtered and evaporated. The yellow solid (1.68 g) was purified by silica gel column chromatography (Merck 230-400 mesh, ϕ = 32 mm, H = 14 cm, *n*-hexane/CH_2Cl_2 = 1:0 ~ 5:1) to obtain a colorless solid (508 mg). The solid was recrystallized from AcOEt to give **1** as colorless crystals (393 mg, 1.30 mmol, 26%).

^1H NMR ($CDCl_3$, 500 MHz, 300 K): δ 7.70 (m, 2H), 7.59 (dt, J = 7.7, 3.9 Hz, 3H), 7.22 (m, 3H), 7.04 (tdd, J = 7.4, 2.0, 1.1 Hz, 2H), 6.57 (dt, J = 8.1, 2.1 Hz, 1H), 3.77 (s, 3H); ^{13}C NMR ($CDCl_3$, 126 MHz, 300 K): δ 160.9, 159.7, 157.7, 145.4 (d, J = 8.2 Hz), 135.3 (d, J = 6.4 Hz), 132.9 (d, J = 35.2 Hz), 132.2 (d, J = 34.0 Hz), 129.4, 126.5, 125.2 (d, J = 11.4 Hz), 113.2, 110.4 (d, J = 11.8 Hz), 55.5; ^{31}P NMR ($CDCl_3$, 202 MHz, 300 K): δ -81.5; mp: 168.5-169.9 °C; FT-IR: 3049.9 (aromatic C-H stretch), 2962.1

(methyl C-H stretch), 1572.7, 1432.8 (aromatic C=C bend), 810.9 (aromatic C-H bend (2), 743.4 (aromatic C-H bend (4)) cm^{-1}; HRMS ($CHCl_3$/CH_3CN/HCO_2H, positive): $[\mathbf{1}\cdot H]^+$ ($C_{19}H_{15}NOP$) *m*/*z* 304.0863 (required, 304.0886).

Supplementary Note 2

Synthesis and single-crystal X-ray analysis of *cis*- and *trans*-$PtCl_2\mathbf{1}_2$

Synthesis of *cis*-$PtCl_2\mathbf{1}_2$

To a 5.0 mM solution of K_2PtCl_4/H_2O (40 mL, 0.20 mmol, 1.0 eq) was added a 10 mM solution of 2-methoxy-9,10-azaphosphatriptycene **1** (0.40 mmol, 2.0 eq) in EtOH (40 mL). The resulting suspended solution was stirred at room temperature for 21 h in the dark. The precipitate was collected by filtration and the residue was washed with H_2O and EtOH, and dried under vacuo to obtain a colorless solid (144 mg). The crude product was purified by reprecipitation from $CHCl_3$/diethyl ether to afford *cis*-$PtCl_2\mathbf{1}_2$ (112 mg, 0.118 mmol, 59%) as a colorless solid.

^{1}H NMR (C_6D_6, 500 MHz, 300 K): δ 7.81-7.78 (m, 4H), 7.71-7.66 (m, 2H), 7.05 (d, J = 7.6 Hz, 4H), 6.83 (s, 2H), 6.31-6.27 (m, 4H), 6.04-6.00 (m, 4H), 5.62-5.68 (m, 2H), 2.57 (s, 2H), 2.55 (s, 4H); ^{31}P NMR (C_6D_6, 202 MHz, 300 K): δ -31.0 ($J_{P\text{-}Pt}$ = 3625 Hz); HRMS ($CHCl_3/CH_3CN$, positive): $[PtCl\mathbf{1}_2]^+$ ($C_{38}H_{28}ClN_2O_2P_2Pt$) *m/z* 836.0958 (required, 836.0957).

Synthesis of *trans*-$PtCl_2\mathbf{1}_2$

A solution of *cis*-$PtCl_2\mathbf{1}_2$ (20.0 mg, 21 μmol) in benzene (20 mL) was irradiated with UV lamp (ASAHI, MAX-303) using a 360 nm filter (band width = 10 nm) at room temperature for 1 h. The solvent was removed by evaporation to obtain a yellow solid (22.5 mg). The crude product was extracted with benzene (2.0 mL) and the resulting yellow suspension was filtered through a membrane filter. Yellow crystals of *trans*-$PtCl_2\mathbf{1}_2$ were obtained by slow evaporation of the benzene solution at room temperature (5.8 mg, 6.1 μmol, 29%).

^{1}H NMR ($CDCl_3$, 500 MHz, 300 K): δ 8.78-8.75 (m, 4H), 8.70 (dt, J = 8.4, 5.4 Hz, 2H), 7.66 (dd, J = 7.7, 1.1 Hz, 4H), 7.34 (td, J = 7.6, 1.2 Hz, 4H), 7.28-7.25 (m, 2H), 7.22 (td, J = 7.5, 1.3 Hz, 4H), 6.72 (dt, J = 8.4, 1.2 Hz, 2H), 3.82 (s, 6H); ^{31}P NMR ($CDCl_3$, 202 MHz, 300 K): δ -39.0 ($J_{P\text{-}Pt}$ = 2937 Hz); HRMS ($CHCl_3/CH_3CN$, positive): $[PtCl\mathbf{1}_2]^+$ ($C_{38}H_{28}ClN_2O_2P_2Pt$) *m/z* 836.0958 (required, 836.0957).

Single-crystal X-ray analysis of *cis*-$PtCl_2\mathbf{1}_2$

Crystals suitable for X-ray analysis of *cis*-$PtCl_2\mathbf{1}_2$·(ether) were obtained by liquid-liquid diffusion of diethyl to a toluene solution of *cis*-$PtCl_2\mathbf{1}_2$ at room temperature.

Crystal data for *cis*-$PtCl_2\mathbf{1}_2$·(ether): $C_{42}H_{38}Cl_2N_2O_3P_2Pt$, Fw = 946.72, colorless, platelet, 0.11 × 0.08 × 0.02 mm^3, monoclinic, space group: $P2_1/c$ (#14), a = 8.19722(18) Å, b = 20.3995(5) Å, c = 23.2204(8) Å, β = 93.0917(16)°, volume: 3877.24(14) Å^3, Z: 4, T = 93 K, λ(MoKα) = 0.71075 Å, $2\theta_{max}$ = 62.2°, R_1 = 0.0852, wR_2 = 0.1491, GOF = 1.125, largest diff. peak and hole: and 1.42/-2.45 e/Å^3. CCDC deposit number 1404948.

Single-crystal X-ray analysis of *trans*-$PtCl_2\mathbf{1}_2$

Crystals suitable for X-ray analysis were obtained by photo-induced isomerization (360 nm) of *cis*-$PtCl_2\mathbf{L}_2$ in benzene at room temperature.

Crystal data for *trans*-$PtCl_2\mathbf{1}_2{\cdot}(C_6H_6)_2$: $C_{50}H_{40}Cl_2N_2O_2P_2Pt$, Fw = 1028.82, colorless, prism, 0.14 × 0.138 × 0.02 mm^3, triclinic, space group P-1 (#2), a = 9.1231(17) Å, b = 11.1245(18) Å, c = 11.509(2) Å, α = 67.404(5)°, β = 75.877(5)°, γ = 76.547(5)°, V = 1033.1(3) $Å^3$, Z = 1, T = 93 K, λ(MoKα) = 0.71075 Å, $2\theta_{max}$ = 50.7°, R_1 = 0.0754, wR_2 = 0.1061 (for all data), GOF = 1.051, largest diff. peak and hole 1.72/-1.87 $eÅ^{-3}$. CCDC deposit number 1404949.

Supplementary Methods

Materials and methods

Unless otherwise noted, solvents and reagents were purchased from TCI Co., Ltd., WAKO Pure Chemical Industries Ltd., Kanto Chemical Co., or Sigma-Aldrich Co., and used without further purification.

^{1}H, ^{13}C, ^{31}P NMR and other 2D NMR spectra were recorded on a Bruker AVANCE III-500 (500 MHz) spectrometer. Tetramethylsilane was used as an internal standard (δ 0 ppm) for ^{1}H and ^{13}C NMR measurements when $CDCl_3$ or mixed solvents containing $CDCl_3$ were used as solvent. A residual solvent signal was used for calibration of ^{1}H NMR measurements when CD_3CN (δ 1.94 ppm) or DMSO-d_6 (δ 2.50 ppm) was used as a solvent.[2] Single-crystal X-ray crystallographic analyses were performed using a Rigaku Saturn724+ diffractometer with MoKα radiation or Rigaku RAXIS-RAPID imaging plate diffractometer with MoKα radiation, and obtained data were calculated using the Crystal Structure crystallographic software package except for refinement, which was performed using SHELXL-2014.[3] All hydrogen atoms were placed geometrically and refined using a riding model. Crystallographic data in this paper can be obtained free of charge from the Cambridge Crystallographic Data Centre (http://www.ccdc.cam.ac.uk/data_request/ cif). ESI-TOF mass data were recorded on a Micromass LCT Premier XE mass spectrometer. Unless otherwise noted, experimental conditions were as follows (Ion mode, positive; Capillary voltage, 3000 V; Sample cone voltage, 30 V; Desolvation temperature, 150 °C; Source temperature, 80 °C). Melting point was measured by Yanaco Micro Melting Point Apparatus MP-500D and uncorrected. Elemental analysis was conducted in the Microanalytical Laboratory, Department Chemistry, Graduate School of Science, the University of Tokyo. IR spectra were recorded on a Jasco FT/IR 4200 with an ATR equipment.

Supplementary References

[1] Malik, Q. M., Ijaz, S., Craig, D. C. & Try A. C. *Tetrahedron* **1997**, *67*, 5798–5805.

[2] Fulmer, G. R., Miller, A. J. M., Sherden, N. H., Gottlieb, H. E., Nudelman, A., Stoltz, B. M., Bercaw, J. E. and Goldberg, K. I., *Organometallics* **2010**, *29*, 2176–2179.

[3] Sheldrick, G. M., *Acta Cryst.* A**64***,* 112–122.
